# Single Nucleotide Polymorphisms Associated with Reading Ability Show Connection to Socio-Economic Outcomes

**DOI:** 10.1007/s10519-017-9859-x

**Published:** 2017-07-15

**Authors:** Michelle Luciano, Saskia P. Hagenaars, Simon R. Cox, William David Hill, Gail Davies, Sarah E. Harris, Ian J. Deary, David M. Evans, Nicholas G. Martin, Margaret J. Wright, Timothy C. Bates

**Affiliations:** 1 Department of Psychology, School of Philosophy, Psychology and Language Sciences, Centre for Cognitive Ageing and Cognitive Epidemiology, The University of Edinburgh, 7 George Square, Edinburgh, EH8 9JZ UK; 2 Division of Psychiatry, University of Edinburgh, Edinburgh, EH10 5HF UK; 3 Medical Genetics Section, University of Edinburgh Centre for Genomic and Experimental Medicine and MRC Institute of Genetics and Molecular Medicine, Western General Hospital, Crewe Road, Edinburgh, EH4 2XU UK; 4 University of Queensland Diamantina Institute, Translational Research Institute, Brisbane, QLD Australia; 5 MRC Integrative Epidemiology Unit, University of Bristol, Bristol, UK; 6 Genetic Epidemiology, QIMR Berghofer Medical Research Institute, Brisbane, Australia; 7 Queensland Brain Institute, University of Queensland, Brisbane, Australia

**Keywords:** Cross-trait linkage disequilibrium regression, MRI, UK Biobank, Polygenic scores, Genetic correlation

## Abstract

Impairments in reading and in language have negative consequences on life outcomes, but it is not known to what extent genetic effects influence this association. We constructed polygenic scores for difficulties with language and learning to read from genome-wide data in ~6,600 children, adolescents and young adults, and tested their association with health, socioeconomic outcomes and brain structure measures collected in adults (maximal N = 111,749). Polygenic risk of reading difficulties was associated with reduced income, educational attainment, self-rated health and verbal-numerical reasoning (p < 0.00055). Polygenic risk of language difficulties predicted income (p = 0.0005). The small effect sizes ranged 0.01–0.03 of a standard deviation, but these will increase as genetic studies for reading ability get larger. Polygenic scores for childhood cognitive ability and educational attainment were correlated with polygenic scores of reading and language (up to 0.09 and 0.05, respectively). But when they were included in the prediction models, the observed associations between polygenic reading and adult outcomes mostly remained. This suggests that the pathway from reading ability to social outcomes is not only via associated polygenic loads for general cognitive function and educational attainment. The presence of non-overlapping genetic effect is indicated by the genetic correlations of around 0.40 (childhood intelligence) and 0.70 (educational attainment) with reading ability. Mendelian randomization approaches will be important to dissociate any causal and moderating effects of reading and related traits on social outcomes.

**Electronic supplementary material:**

The online version of this article (doi:10.1007/s10519-017-9859-x) contains supplementary material, which is available to authorized users.

Disabilities in reading and language are often detected late in childhood, and are a major source of disadvantage not only in formal education (Young et al. 2002; Richardson and Wydell 2003) but beyond (Ritchie and Bates 2013). For example, lower occupational status (Maughan 1995), greater psychological distress (Boetsch et al. 1996), and over-representation in the prison system (Svensson 2011) are all associated with poor acquisition of reading. Whether genes underlie the relationship between childhood reading or language abilities and adult life outcomes is not known because there are no longitudinal family studies which span such a long time. The advent of polygenic scores, based on thousands of small molecular genetic effects influencing a trait, allows unprecedented access to genetic pathways because it does not require data collection of the related traits of interest in the same sample. We utilise this method to test the association between genes influencing reading and language traits in development and measures of educational attainment, income, self-rated health, depression, handedness and brain magnetic resonance imaging (MRI) in mid-to-late adulthood.

Variation in reading and language abilities is influenced by genetic and environmental determinants (Defries et al. 1987; Bates et al. 2007; Kovas et al. 2005; Hayiou-Thomas 2008), with the same genetic factors appearing to be relevant for ability and disability (Harlaar et al. 2005; Haworth and Plomin 2010; Lind et al. 2010). These data, largely from twin samples, have been supplemented by major advances in molecular genetic analysis, revealing common genetic variants (i.e., Single Nucleotide Polymorphism or SNPs) in or near genes (e.g., *KIAA0319*) affecting neuronal migration and raising risk for developmental communication disorders (Meng et al. 2005; Scerri et al. 2011). The era of low-cost genome-wide association (GWA) studies has further contributed to understanding of complex traits, not only with new candidates, but also as a basis to build polygenic risk scores. This method takes advantage of the fact that, independent of the stringent criteria for significance of evidence for association (which with small samples and small effects will fall well short of the genome-wide significance criterion), the individual effect weights for each of the thousands of independent SNPs may be summed to form an estimate of risk. These scores may be used to predict the same trait in fully-independent samples, or as a linking function, to evaluate relationships of the trait with other outcomes (Evans et al. 2009). It is this latter function which we exploit here.

Genome-wide association studies of reading and language traits indicate that, like most psychological traits, they are genetically complex: that is, communication is influenced by many thousands of gene differences, each of very small individual effect. Even among the candidate genes for reading and language that have received the most support (e.g., *DCDC2, KIAA0319*), effect sizes do not typically exceed 1% (Lind et al. 2010; Francks et al. 2004). GWA studies of reading and language traits indicate that the majority of novel association loci will have effect sizes less than 0.4% (Meaburn et al. 2008; Gialluisi et al. 2014; Luciano et al. 2013). Polygenic scores, which combine information from SNP effects on a trait, allow us to study the effects of genes influencing a trait without requiring each individual SNP to be significant. Such a method was effectively used, for instance, by Belsky and colleagues (2016) to study the relationship of a polygenic score for higher educational attainment with social mobility and adult economic status. Their polygenic scores for educational attainment were based on results from a very large GWA study of around 126,000 individuals, although their independent prediction sample included only 1037 participants.

In the present study, the sample used to generate the polygenic scores for reading and language is modest, although similar-sized GWA studies have successfully predicted cross-trait variation. For instance, polygenic neuroticism (based on a GWA sample of 6268) predicted depressive symptoms (Luciano et al. 2012) and polygenic autism spectrum disorder (GWA N ~6700) predicted cognitive abilities (Clarke et al. 2016). Less reliable polygenic scores will limit the variance that can be explained in a trait, but the sample we predict into is extremely large, with up to 111,785 participants from UK Biobank, giving us sufficient power to detect the smaller expected effect sizes. Importantly, the GWA study on which we base our reading and language polygenic scores controlled for the effects of non-verbal IQ, so any associations that we uncover should be independent of the effects of intelligence, which is also known to associate with various adult outcome measures of interest. To confirm this, genetic correlations between reading and language traits with childhood intelligence are estimated. Successful prediction by polygenic reading and language scores indirectly validates the results from the prior GWAS on which the scores are based. The results further complement those from GWAS of broader traits, like IQ or educational attainment, because the polygenic score can be tied more specifically to the narrowly defined and measured trait.

## Methods

### Participants

Participants were drawn from the UK Biobank (http://www.ukbiobank.ac.uk), an open resource enabling the study of factors influencing disease in mid- to late (40–69 years) adulthood (Sudlow et al. 2015). The baseline survey measures (including questionnaire and biological samples) were gathered online and in specific locations in the UK between 2006 and 2010 on 502,655 community residing individuals. At baseline, measures of income, education, self-rated health, depression, handedness, and cognitive ability (reasoning, reaction time) were relevant to the present study (N varied 18,321–111,749). Between 2014 and 2015, a web-based questionnaire, completed remotely, obtained data for a further two cognitive tests (trail making and symbol digit substitution) on a subsample of 23,757 and 26,914, respectively. MRI brain scans were available for a subset of participants (N = 5455), who were imaged around 4 years after initial recruitment. Only a subset of these passed quality control of MRI data and had genetic array data (maximum of N = 1206). DNA was extracted from blood samples and genotyping performed with either the UK BiLEVE array or the UK Biobank axiom array. Standard quality control procedures, including exclusions for gender mismatch and non-British ancestry, were applied. Further description can be found in (Hagenaars et al. 2016). PRS were calculated using the observed genotypes. The recoding from numeric (1, 2) allele coding to standard ACGT format is described in (Hagenaars et al. 2016). UK Biobank received ethical approval from the Research Ethics Committee (REC reference 11/NW/0382). This study has been completed under UK Biobank application 10,279.

## Measures

### Socio-economic

#### Income

The household average total income before tax was measured on a five-point scale: <£18,000; £18,000 to £30,999; £31,000 to £51,999; £52,000 to £100,000; >£100 000. ‘Do not know’ (N = 4319) and ‘Prefer not to answer’ (N = 10,553) responses were coded as missing.

#### Education

Participants were asked to report their qualifications using six categories, with multiple responses being allowed—College or University Degree; Advanced level- or Advanced Supplementary-levels or equivalent; Ordinary-levels (or General Certificate of Secondary Education or equivalent); Certificate of Secondary Education or equivalent; National Vocational Qualifications or Higher National Diploma or Higher National Certificate or equivalent; and Other professional qualifications e.g. nursing, teaching. A binary variable was constructed for use in analysis: college/university degree versus none.

### Cognitive

#### Verbal-numerical reasoning

Thirteen logic/reasoning-type questions were given, each with a two-minute time restriction. The sum-score of correct response was used as our dependent variable. In the UK Biobank protocol (http://biobank.ctsu.ox.ac.uk/crystal/field.cgi?id=20016) this variable is labelled ‘fluid intelligence’ although it includes verbal and numerical content. The variable had reasonable internal reliability (Cronbach alpha coefficient of 0.62) given the mixture of item types and test–retest reliability (*r* = 0.65) over an average 4-year interval (Hagenaars et al. 2016; Lyall et al. 2016).

#### Reaction time

Reaction time (RT) was measured by the speed with which the participant pressed a button in response to a pair of matching symbols. It was described to the participant as akin to the card game ‘Snap’ (http://biobank.ctsu.ox.ac.uk/crystal/field.cgi?id=20023). Mean response time in milliseconds across correct trials was the dependent variable. The task has shown good internal reliability with a Cronbach’s alpha of 0.85, but lower test–retest reliability (*r* = 0.57) over a mean 4-year interval in UK Biobank (Hagenaars et al. 2016; Lyall et al. 2016).

#### Trail making

This is a computer adapted version of the Trail making test that has two parts: A and B. Trails A required the participant to quickly and accurately link numbers appearing in circles on the screen in numeric order; they do this using the computer mouse, or touchpad of laptop or tablet. In Trails B, numbers and letters are presented in the circles, and the participant is required to alternate between linking numbers in numeric order and letters in alphabetical order. The time taken to correctly link the circles is the dependent variable, and here we focus on Trails B, which reflects executive functioning.

#### Symbol digit

In this processing speed task, participants are shown a key at the top of the screen in which eight symbols are paired with the numbers one to eight. Below this key test items are presented in a series of grids containing symbols which require a matched numeric response using the key. Responses are made by clicking on the number pad below the grid, with participants instructed to complete the grid from left to right as quickly as they can within the specified 2-min time period. The dependent variable is the number of correct items.

#### Handedness

Participants were asked whether they were right- or left-handed; ambidextrous individuals were excluded from the analysis. Coding was in the positive direction of left-handers.

### Health

#### Self-rated health

On a four-point scale, participants were asked to rate their overall health as excellent, good, fair or poor, therefore, higher scores reflected poorer health.

#### Depression

The experience of depression, both previous and current, was assessed by asking questions about lifetime experience of minor and major depression as detailed online in the UK Biobank touchscreen questionnaire. Further items from the Patient Health Questionnaire (PHQ: Spitzer et al. 1999) and history of help-seeking for mental health problems was used to categorise depression into recurrent episode status as used in the present study. Details on the classification procedure are contained in Smith et al. (2013); diagnostic criteria were arrived at by agreement of 12 mental health/cognition researchers and aligned as much as possible with the International Classification of Diseases (ICD-10) and the American Psychiatric Association’s Diagnostic and Statistical Manual (DSM-IV). The following items were relevant for classification: “Looking back over your life, have you ever had a time when you were feeling depressed or down for at least a whole week?”; “Have you ever had a period of time lasting at least 2 days when you were so irritable that you found yourself shouting at people or starting fights or arguments?”; “How many weeks was the longest period when you were feeling depressed or down?”; “How many periods have you had when you were feeling depressed or down for at least a whole week?”; “Have you ever seen a general practitioner (GP) for nerves, anxiety, tension or depression?”; “Have you ever seen a psychiatrist for nerves, anxiety, tension or depression?”

### Brain magnetic resonance imaging (MRI)

Data were acquired on a single standard Siemens Skyra 3T scanner with a standard Siemens 32-channel RF receiver head coil, with the imaging matrix at a downward angle of 16° from the AC-PC line. All data were acquired, processed, quality checked and distributed by UK Biobank as Imaging Derived Phenotypes (IDPs). Full information is publicly accessible on the UK Biobank website in the form of a Protocol (http://biobank.ctsu.ox.ac.uk/crystal/refer.cgi?id=2367), Brain Imaging Documentation (http://biobank.ctsu.ox.ac.uk/crystal/refer.cgi?id=1977) alongside further processing pipeline information (Miller 2016). In short, T1-weighted volumes were acquired using a 3D MPRAGE sagittal sequence at 1 mm^3^ resolution with a 208 × 256 × 256 field of view (FoV). For diffusion MRI, a spin-echo echo-planar imaging (SE-EPI) sequence with 10 T2-weighted (b ≈ 0 s mm^−2^) baseline, 50 b = 1000 s mm^−2^ and 50 b = 2000 s mm^−2^ diffusion-weighted volumes acquired with 100 distinct diffusion-encoding directions and three times multi-slice acquisition was used. 2 mm isotropic voxels were provided with a field of view of 104 × 104 mm, imaging matrix 52 × 52, 72 slices with slice thickness 2 mm. Volumetric and diffusion MRI and tractography processing details can also be found in Cox et al. (2016). For brain volume, we focus on grey matter, white matter and total brain volume; these have been corrected for head size by using a SIENAX-type procedure which applies a scaling factor to the volumes that had been extracted from the normalisation transform matrix resulting from the affine registration of skull tissue between T1-weighted volume and MNI152 space. For diffusivity, we focus on measures of fractional anisotropy (FA) and mean diffusivity (MD) in available regions of interest that have been previously tied to reading and/or language: the inferior longitudinal fasciculus and superior longitudinal fasciculus in separate left and right hemispheres (Elnakib et al. 2014; Vandermosten et al. 2012). Following correction for gradient distortion (http://github.com/Washington-University/Pipelines), head motion and eddy currents (http://fsl.fmrib.ox.ac.uk/fsl/fslwiki/EDDY), the within-voxel tract orientation structure was modelled using BEDPOSTx, followed by probabilistic tractography (using PROBTRACKx: Behrens et al. 2007). Tract-averaged measures of FA and MD were then derived using automatic tract mapping with the AutoPtx plug-in for FSL (de Groot et al. 2013). Participant data was excluded from the present analysis if they reported, at contemporaneous medical interview, any of the following conditions: of dementia, Parkinson’s disease or any other chronic degenerative neurological problem (including demyelinating diseases), brain cancer, brain haemorrhage, brain abscess, aneurysm, cerebral palsy, encephalitis, head injury, nervous system infection, head or neurological injury or trauma, or stroke.

### Statistical analysis

Polygenic scores were based on genome-wide meta-analysis results (Luciano et al. 2013) for two measures of reading (word reading, general reading and spelling component) and one measure of language (non-word repetition). The meta-analysis was based on two samples: the Brisbane Adolescent Twin Study (BATS; N = 1177, age range 12–26 years, mean of 18 years for reading tests and 20 for language test) and the Avon Longitudinal Study of Parents and their Children (ALSPAC; N = 5472, aged 8 years for language test and 9 years for reading test). All reading and language measures were controlled for the effects of non-verbal (performance) IQ in the genome-wide analyses. Polygenic scores for childhood (6–18 years) general intelligence (Benyamin et al. 2014) and adult educational attainment (college degree) (Rietveld et al. 2013) were further created in UK Biobank based on the largest publicly available GWA studies estimated in respective sample sizes of 17,989 and 101,069. They were used to test whether reading and language polygenic scores effects were independent from the polygenic influences on general cognitive ability, and, as expected, whether they correlated with genetic influences on educational attainment.

Using PRSice software (Euesden et al. 2015), five polygenic scores were created for each variable of interest based on the significance value of each SNP from the GWA meta-analysis: p < 0.01, p < 0.05, p < 0.1, p < 0.5, and p < 1. We focus on the results of polygenic scores based on all SNPs that perform as well as or better than scores based on smaller sets (Dudbridge 2013), but all results can be found in the online supplementary material. Prior to calculating the scores, exclusions were made for: low minor allele frequency (<0.01) SNPs, and SNPs in linkage disequilibrium (r^2^ > 0.25) using a clumping method within a 250 kb window. The clumping method preferentially selects SNPs showing the greatest association for the variable of interest. For the word reading polygenic scores, calculations were based on 134,035 SNPs, reading and spelling component polygenic scores on 134,315 SNPs, and non-word repetition polygenic scores on 134,266 SNPs. The regression models for these polygenic scores predicting the range of adult outcome variables included additional covariates: age at survey, sex, genotyping batch and array, assessment centre, and the first ten genetic principal components (to correct for population stratification). False discovery rate correction was applied to the 300 tests (i.e., 3 polygenic reading and language traits at 5 threshold levels × 20 phenotypic traits). For any significant tests, a further analysis including childhood intelligence polygenic scores as a predictor were performed to confirm whether the reading/language polygenic effects were independent of those influencing general cognitive ability.

Pearson’s correlations between the reading and language polygenic scores with cognitive ability and educational attainment polygenic scores were estimated and genetic correlations were derived by a cross-trait linkage disequilibrium score regression method as described in Bulik-Sullivan et al. (2015). This latter method only relies on GWA summary statistics and is not biased by sample overlap.

## Results

Data distributions were checked for normality (log transformation applied to RT) and extreme scores identified and removed [0.05% for log RT (long RTs), 0.23% for symbol digit (zero scores possibly indicating non-comprehension of the task) and 0.27% for trail making (long RTs)]. The descriptive statistics for the adult socio-economic, health, cognitive, and MRI traits in UK Biobank are shown in Table S1 in the Supplemental Material online.

The correlations among the reading and language polygenic scores were generally lower at the more restrictive polygenic inclusion levels. At the p < 0.01 SNP inclusion level, the non-word repetition polygenic score showed correlations of ~0.05 with the two reading polygenic scores, increasing to ~0.55 when the SNP inclusion threshold was p < 1. Correlations between the two reading polygenic scores ranged 0.47 at the p < 0.01 SNP inclusion level to 0.77 at the p < 1 SNP inclusion level. In comparison, at the p < 1 SNP inclusion level, the childhood intelligence polygenic score correlated 0.094 with the reading and spelling component polygenic score, 0.063 with the word reading polygenic score, and 0.093 with the non-word repetition polygenic score (all p = 2.2 × 10^−16^). Educational attainment polygenic scores correlated 0.053 with the reading and spelling component, 0.036 with word reading, and 0.043 with non-word repetition polygenic scores (all p = 2.2 × 10^−16^). Genetic correlations, estimated by linkage disequilibrium regression, between the reading and language measures with childhood intelligence and educational attainment are shown in Table 1. Despite observed limitations in power, a significant genetic correlation was shown for the reading and spelling component and childhood intelligence (0.40) and for all measures with educational attainment (ranging 0.56 for non-word repetition to 0.78 for word reading).

**Table 1:** Genetic correlations between the reading and language traits with childhood intelligence and educational attainment

Phenotypes	Genetic correlation	Standard error	*P* value	Heritability Z-score	Mean χ^2^
Childhood intelligence					
Reading and spelling component	0.401	0.174	0.021	2.507	1.046
Word reading	0.302	0.200	0.131	1.808	1.029
Non-word repetition	0.527	0.319	0.098	1.093	1.039
Educational attainment					
Reading and spelling component	0.699	0.144	1.130 × 10^− 6^	3.118	1.043
Word reading	0.776	0.192	5.236 × 10^− 5^	2.244	1.028
Non-word repetition	0.556	0.198	4.919 × 10^− 3^	1.562	1.035

The heritability Z-score and the mean χ^2^ indicate the level of power to detect association where a heritability Z-score of >4 and a mean χ^2^ > 1.02 being considered well powered (Bulik-Sullivan et al. 2015)

The associations between the three polygenic scores (word reading, reading and spelling component, non-word repetition) and the socio-economic, health, cognitive, and MRI traits are shown in Table 2. Regression betas for polygenic scores based on more restrictive SNP inclusion criteria can be found in Table S2 in the Supplemental Material online. 56 of the 300 tests were significant at a nominal p < 0.05 level; FDR correction supported significant association for 24 of these. For the associations reported in Table 2, the reading and spelling component polygenic score significantly predicted educational attainment, income, self-rated health, and verbal-numerical reasoning. The word reading polygenic score was significantly associated with verbal-numerical reasoning. Associations between polygenic scores and the brain MRI phenotypes were of comparable magnitudes, but were available in a much reduced sample, and we did not reliably detect any significant associations. The results for the most consistently associated variables—income, educational attainment, self-rated health, and verbal-numerical reasoning—at all polygenic score SNP inclusion levels are graphically depicted in Figure S1 in the Supplemental Material online.

**Table 2:** Standardised betas from the regression of socio-economic, health, cognitive and brain MRI traits on the reading and language polygenic scores

Phenotypes	N	Word reading		Reading and spelling component		Non-word repetition	
		*Beta*	*p*	*Beta*	*p*	*Beta*	*p*
SES							
Income	96,900	**0.008**	**0.006**	**0.011**	**0.0003**	**0.006**	**0.038**
College/university	111,114	**0.009**	**0.002**	**0.018**	**5.67 × 10** ^**−10**^	**0.010**	**0.001**
Health							
Self-rated health	111,749	−0.005	0.089	−**0.012**	**9.49 × 10** ^**− 5**^	−**0.009**	**0.002**
Depression recurrent	18,321	− 0.005	0.529	0.001	0.889	0.002	0.738
Cognitive							
Verbal-numerical	36,035	**0.019**	**0.0004**	**0.032**	**7.04 × 10** ^**− 10**^	**0.013**	**0.011**
Reaction time	111,425	0.002	0.518	0.000	0.863	0.0004	0.885
Symbol digit	26,914	0.001	0.806	0.004	0.491	−0.001	0.877
Trails B	23,757	−0.004	0.523	−0.005	0.369	−0.004	0.050
Handedness	110,375	0.002	0.562	0.004	0.199	0.002	0.397
MRI traits							
Fractional anisotropy							
Left ILF	1 049	0.048	0.111	0.047	0.120	0.003	0.917
Right ILF	1 049	0.029	0.338	0.034	0.254	−0.007	0.803
Left SLF	1 049	0.026	0.384	0.048	0.108	0.025	0.401
Right SLF	1 049	0.015	0.624	0.061	0.043	0.037	0.216
Mean diffusivity							
Left ILF	1 049	−0.046	0.118	−0.052	0.081	−0.005	0.857
Right ILF	1 049	−0.028	0.330	−0.026	0.381	0.002	0.944
Left SLF	1 049	−0.034	0.249	−0.031	0.301	0.016	0.584
Right SLF	1 049	−0.018	0.544	−0.027	0.364	0.014	0.637
Volumes							
Grey matter	1 206	−0.022	0.332	0.000	0.989	−0.036	0.107
White matter	1 206	−0.003	0.918	0.016	0.573	0.007	0.811
Total brain	1 206	−0.016	0.522	0.009	0.714	−0.020	0.422

The models include adjustment for standard covariates. All nominally significant results (p < 0.05) are in bold typeface, although FDR significance was at a p < 5.55 × 10^− 4^

*ILF* inferior longitudinal fasciculus, *SLF* superior longitudinal fasciculus

For the reading and spelling component polygenic associations that withstood FDR correction, further regression analyses which adjusted for childhood intelligence polygenic effects (Table 3), and additionally, educational attainment polygenic effects (Table 4) were performed. The results in Table 3 show that the reading and spelling component polygenic effects remained significant although they were smaller than those due to polygenic childhood intelligence. A sensitivity analysis which adjusted for a further five population stratification components (to confirm stability of the parameters) left these betas unchanged. Additional adjustment for educational attainment showed further weakening of the reading and spelling polygenic associations, with the income association no longer significant. The educational attainment polygenic score explained more variance in the adult outcomes than both the reading ability and childhood intelligence polygenic predictors.

**Table 3:** Standardised betas from the regression of socio-economic, health, and cognitive traits on the reading and spelling component and childhood intelligence polygenic scores

		SES		Health	Cognitive
		Income (N = 96,900)	College/university (N = 111,114)	Self-rated health (N = 111,749)	Verbal-numerical reasoning (N = 36,035)
Polygenic score					
Reading and spelling component	*Beta*	0.008	0.013	−0.009	0.025
	*p*	0.008	5.95 × 10^− 6^	0.002	1.47 × 10^− 6^
Childhood intelligence	*Beta*	0.030	0.053	−0.024	0.076
	*p*	2 × 10^− 16^	2 × 10^− 16^	2.68 × 10^− 16^	2 × 10^− 16^

The model includes adjustment for standard covariates

A sensitivity analysis including an additional 5 population stratification components as covariates produced no change in Beta values, with minor increases in p-values

**Table 4:** Standardised betas from the regression of socio-economic, health, and cognitive traits on the reading and spelling component, childhood intelligence and educational attainment polygenic scores

		SES		Health	Cognitive
		Income (N = 96,900)	College/university (N = 111,114)	Self-rated health (N = 111,749)	Verbal-numerical reasoning (N = 36,035)
Polygenic score					
Reading and spelling component	*Beta*	0.005	0.009	−0.007	0.022
	*p*	0.067	0.002	0.012	3.65 × 10^− 5^
Childhood intelligence	*Beta*	0.025	0.045	−0.021	0.07
	*p*	2 × 10^− 16^	2 × 10^− 16^	6.91 × 10^− 12^	2 × 10^− 16^
Educational attainment	*Beta*	0.071	0.120	−0.053	0.095
	*p*	2 × 10^− 16^	2 × 10^− 16^	2 × 10^− 16^	2 × 10^− 16^

The model includes adjustment for standard covariates

## Discussion

We examined how genetic risk for reading and language difficulties, assessed using polygenic scores, may, independent of intelligence, influence consequential adult outcomes such as income and health. The major finding was that, expressed in positive terms, polygenic scores acting to increase reading ability were significantly associated with higher educational attainment, higher incomes and greater self-rated health as well as with higher verbal-numerical reasoning. Low to moderate genetic correlations between reading traits and childhood intelligence were found, but accounting for polygenic childhood intelligence effects did not significantly alter the polygenic reading associations with adult outcomes. These data might reflect a causal role of the pathway from reading to long-term social outcomes, consistent with a report of reading and language skills mediating the genetic effects on these social outcomes (Ritchie and Bates 2013; Ritchie et al. 2013). The present study could not address causality, but given that reading and language skills are developed early in life a causal direction from these abilities to later life outcomes is a tenable hypothesis if one assumes no genetic confounding from factors like parental care and socio-economic status. The polygenic reading ability associations were reduced when polygenic educational attainment was controlled for in the models. This suggests that shared genetic influences on reading ability and adult outcomes are, in part, mediated by genetic effects on educational attainment. Indeed, the strong genetic correlation between reading and educational attainment indicate a dependence on common genes. However, some unique predictive power of polygenic reading scores remained for education, self-rated health and, especially, verbal-numerical reasoning in these models. The shared polygenic variance possibly related to genetic effects influencing reading ability that are independent of the polygenic load associated with childhood intelligence, and educational attainment.

Ritchie and colleagues (2013) found that higher reading achievement at age seven was associated with higher socio-economic status at age 42 in women independent of its effects on intelligence at age 11, academic motivation at age 16 and years of education (these indirect effects were significant in men and women). Given that the reading polygenic effects we tested were independent of childhood intelligence polygenic effects, the correlation between polygenic reading scores and income could reflect such a direct effect between reading and income or an indirect effect mediated by motivation and years of education [consistent with the moderate genetic correlation observed between reading and educational attainment and also consistent with the large genetic correlation, r_g_ = 0.90, between the number of years spent in education and household income (Hill et al. 2016)]. A larger effect might be expected for a measure of individual’s income rather than the household measure available in the present study. The association we found between polygenic reading scores and verbal-numerical reasoning might also reflect a direct process given that the effect was present when childhood intelligence polygenic scores were also in the model. This is consistent with non-shared environmental effects on reading ability that lead to variation in later intelligence (Ritchie et al. 2015), although in this case it manifests as genetic variation.

In terms of causal mechanism, one possibility is that a gene-environment correlation is operating such that those who are genetically predisposed to a higher reading ability are able to better engage with stimuli present in the educational setting and indeed seek out more challenging cognitive endeavours that propels their learning and resultant cognitive abilities, and in turn, educational attainment. The moderate to strong genetic correlations between reading and language traits with educational attainment suggest that reading and language skills are contributors to genetic variation in education attainment. That is, additive genetic effects do not act directly on educational attainment but on those factors (such as reading skill) that influence whether one completes college or not. There was no association between polygenic scores with processing speed or executive function, which might suggest that the advantage that genes influencing reading confer are on abilities that are largely dependent on culture (within UK Biobank verbal-numerical reasoning is arguably a measure of crystallized intelligence; Hagenaars et al. 2016). Alternatively, the null findings for the other tests might be due to their poor psychometric properties (Lyall et al. 2016).

With regard to health, correlational studies in children and young adults have shown that the presence of dyslexia or reading difficulties is associated with greater mood disturbance, like depressive symptoms (Mugnaini et al. 2009). Our findings show that this association is not due to genetic overlap, at least for any enduring relationship between early reading ability to depression in mid-life and later. It is possible that over time, children with reading and/or language difficulties develop strategies to overcome the associated life challenges that might otherwise trigger depression. Notwithstanding, we find no support for genes influencing reading ability being related to recurrent depression. A systematic review (Dewalt et al. 2004) found that reading ability was related to hospitalisation, general indices of health, various chronic ailments and knowledge concerning health. Two large studies in the US showed an association between low literacy and poor self-reported health (Baker et al. 1997; Gazmararian et al. 1999); in one of these, the effect of literacy was greater than years of education (Baker et al. 1997). In our study, the reading and spelling component polygenic score was correlated with self-rated health, in the same direction as previous phenotypic reports. One might speculate that this is again an instance of mediated pleiotropy where reading ability affects one’s health literacy, resultant care, and self-reported health. The genetic correlation between years of education and self-rated health in UK Biobank was r_g_ = 0.59 (Harris et al. 2016), it is possible that part of this covariance is due to polygenic influences on reading ability.

Research shows that brain indices of white matter structural connectivity, particularly in a region including the left arcuate fasciculus and the left corona radiata (Vandermosten et al. 2012), and of grey and white matter volumes (Elnakib et al. 2014) differ between dyslexics and controls. Furthermore, genetic risk variants for reading impairment have been linked to various imaging variables (Eicher and Gruen 2013). Despite this, our study found no associations between genes influencing reading and language traits with MRI variables or handedness, potentially linked to brain asymmetry. However, the sample size for the MRI analyses (maximum N of 1206) was much smaller than the other analyses (which were at least 20 times larger); notably, the effect sizes for MRI traits were larger. Characteristics of cortical regions, such as volume, surface area and thickness, may also offer valuable insights but are currently not available in this sample. Future work on focussed regions associated with reading and language should be a priority.

Our effect sizes were small with one standard deviation change in reading polygenic scores equal to at most a 0.03 of a standard deviation change in verbal-numerical reasoning. The same effects for polygenic childhood intelligence and education attainment were around three times larger, but this was expected given that verbal-numerical reasoning is an index of general cognitive ability and that educational attainment is largely determined by one’s general cognitive ability. The much larger GWA samples on which the latter two scores were based would have also contributed to their better prediction. That we could detect polygenic reading ability effects for various socioeconomic, cognitive, and health measures, suggests that even relatively small GWA samples can capture genetic signal (albeit weak) for reading ability. Nevertheless, much larger GWA studies of reading and language traits are needed to confirm the size and distribution of individual gene effects and to produce more robust polygenic scores which will predict greater variance. They will also allow sufficient statistical power to confirm genetic correlations with other traits, which our study was underpowered to do. Observationally, the lower genetic correlations between reading ability and childhood intelligence than with adult intelligence (via the proxy, educational attainment) suggest that with development reading ability and intelligence (or at least academic achievement) become coupled. Language ability did not show this difference. Given that the reading and language ability GWA analyses controlled for non-verbal IQ, the genetic correlations could be indicative of direct effects of reading/language on intelligence and educational attainment rather than the reverse or pleiotropy.

Our study has demonstrated that the genes influencing reading ability in childhood through to young adulthood affect later adult outcomes related to social status, education and health. These may operate via a causal process from reading ability to adult outcome, via an unmeasured variable (e.g., motivation) affecting both reading ability and adult outcome, or as direct pleiotropic gene effects on reading ability and adult outcome. Mendelian randomization studies may be a way forward to disentangle the unresolved causal relationships reflected in this genetic overlap, although it will be important to model known mediators or use a multi-phenotype approach.

## Electronic supplementary material

Below is the link to the electronic supplementary material.

Supplementary material 1 (DOCX 20 KB)

Supplementary material 2 (XLSX 17 KB)

Supplementary material 3 (DOCX 348 KB)
