# Supplementary material for: Single Nucleotide Polymorphisms Associated with Reading Ability Show Connection to Socio-Economic Outcomes
Source: Behav Genet. 2017 Jul 15;47(5):469–79. doi: 10.1007/s10519-017-9859-x (PMC5574963; doi:10.1007/s10519-017-9859-x)
Supplement: Supplementary file 1 — Supplementary material 1 (DOCX 20 KB) [file 10519_2017_9859_MOESM1_ESM.docx]

Table S1 Mean and frequencies for the socio-economic, health, cognitive, and MRI traits in UK Biobank

|  |  | Total | |  | Women | |  | Men | |
| --- | --- | --- | --- | --- | --- | --- | --- | --- | --- |
|  | N | Mean/Frequency | SD | N | Mean/Frequency | SD | N | Mean/Frequency | SD |
| Age |  | 56.91 | 7.93 | 58,914 | 56.6 | 7.83 | 53,237 | 57.25 | 8.02 |
| **SES** |  |  |  |  |  |  |  |  |  |
| Household Income  <£18,000  £18,000–£30,999  £31,000–£51,999  £52,000–£100,000  > £100,000 | 96,900 | 22,145 (22.8%)  25,061 (25.9%)  25,613 (26.4%)  19,247 (19.9%)  4 834 (5%) |  | 49,076 | 12,319 (25.1%)  13,092 (26.7%)  12,588 (25.6%)  8 901 (18.1%)  2 176 (4.4%) |  | 47,824 | 9 826 (20.5%)  11,969 (25%)  13,025 (27.2%)  10,346 (21.6%)  2 658 (5.5%) |  |
| College/University  Yes  No | 111,114 | 33,852 (30.5%)  77,262 (69.5%) |  | 58,386 | 17,118 (29.3%)  41,268 (70.7%) |  | 52,728 | 16,734 (31.7%)  35,994 (68.3%) |  |
| **Health** |  |  |  |  |  |  |  |  |  |
| Self-Rated Health  Excellent  Good  Fair  Poor | 111,749 | 18,202 (16.3%)  64,567 (57.8%)  23,897 (21.4%)  5 083 (4.5%) |  | 58,913 | 10,000 (17%)  34,873 (59.4%)  11,504 (19.6%)  2 336 (4%) |  | 53,237 | 8 202 (15.5%)  29,694 (56%)  12,393 (23.4%)  2 747 (5.2%) |  |
| Depression Recurrent  Yes  No | 18,321 | 3 923 (21.4%)  14,398 (78.6%) |  | 9250 | 2 470 (26.7%)  6 780 (73.3%) |  | 9071 | 1 453 (16%)  7 618 (84%) |  |
| **Cognitive** |  |  |  |  |  |  |  |  |  |
| Verbal-numerical | 36,035 | 6.16 | 2.10 | 18,771 | 6.07 | 2.04 | 17,264 | 6.26 | 2.17 |
| Reaction Time | 111,425 | 6.30 | .18 | 58,549 | 6.31 | .18 | 52,876 | 6.28 | 0.18 |
| Symbol digit | 26,914 | 19.7 | 5.13 | 14,631 | 19.94 | 5.25 | 12,283 | 19.49 | 4.98 |
| Trails B | 23,757 | 66.09 | 23.85 | 12,703 | 66.28 | 23.74 | 11,054 | 65.88 | 23.98 |
| Handedness  Left  Right | 110,375 | 10,764 (9.8%)  99,611 (90.2%) |  | 58,221 | 5 084 (8.7%)  53,137 (91.3%) |  | 52,154 | 5 680 (10.9%)  46,474 (89.1%) |  |
| **MRI Traits** |  |  |  |  |  |  |  |  |  |
| *Fractional Anisotropy* |  |  |  |  |  |  |  |  |  |
| Left ILF | 1 049 | .46 | .02 | 542 | .46 | .02 | 507 | .46 | .02 |
| Right ILF | 1 049 | .45 | .02 | 542 | .45 | .02 | 507 | .45 | .02 |
| Left SLF | 1 049 | .44 | .02 | 542 | .44 | .02 | 507 | .44 | .02 |
| Right SLF | 1 049 | .43 | .02 | 542 | .42 | .02 | 507 | .43 | .02 |
| *Mean Diffusivity* |  |  |  |  |  |  |  |  |  |
| Left ILF | 1 049 | .0008 | .00003 | 542 | .0008 | .00003 | 507 | .0008 | .00003 |
| Right ILF | 1 049 | .0008 | .00003 | 542 | .0008 | .00003 | 507 | .0008 | .00003 |
| Left SLF | 1 049 | .0007 | .00003 | 542 | .0007 | .00003 | 507 | .0007 | .00003 |
| Right SLF | 1 049 | .0007 | .00003 | 542 | .0008 | .00003 | 507 | .0007 | .00003 |
| *Volumes* |  |  |  |  |  |  |  |  |  |
| Grey Matter | 1 206 | 797,199 | 47,935 | 624 | 813,530 | 44,957 | 582 | 779,689 | 44,775 |
| White Matter | 1 206 | 711,301 | 41,796 | 624 | 708,795 | 41,347 | 582 | 713,987 | 42,143 |
| Total Brain | 1 206 | 1,508,500 | 73,988 | 624 | 1,522,325 | 72,419 | 582 | 1,493,677 | 72,832 |

Note: ILF: Inferior Longitudinal Fasciculus; SLF: Superior Longitudinal Fasciculus
