# Supplementary material for: Single Nucleotide Polymorphisms Associated with Reading Ability Show Connection to Socio-Economic Outcomes
Source: Behav Genet. 2017 Jul 15;47(5):469–79. doi: 10.1007/s10519-017-9859-x (PMC5574963; doi:10.1007/s10519-017-9859-x)

Figure S1. Reading and language polygenic scores (NWR: non-word repetition; PC: reading and spelling principal component; Reading: word reading) at five SNP inclusion thresholds (x-axis) predicting educational attainment, household income, self-rated health, and verbal-numerical reasoning in UK Biobank. Standardised betas for the polygenic score effect are depicted on the y-axis and the significance value of the polygenic predictor is displayed on the bars (p = 0 represents a p-value < 0.0001).


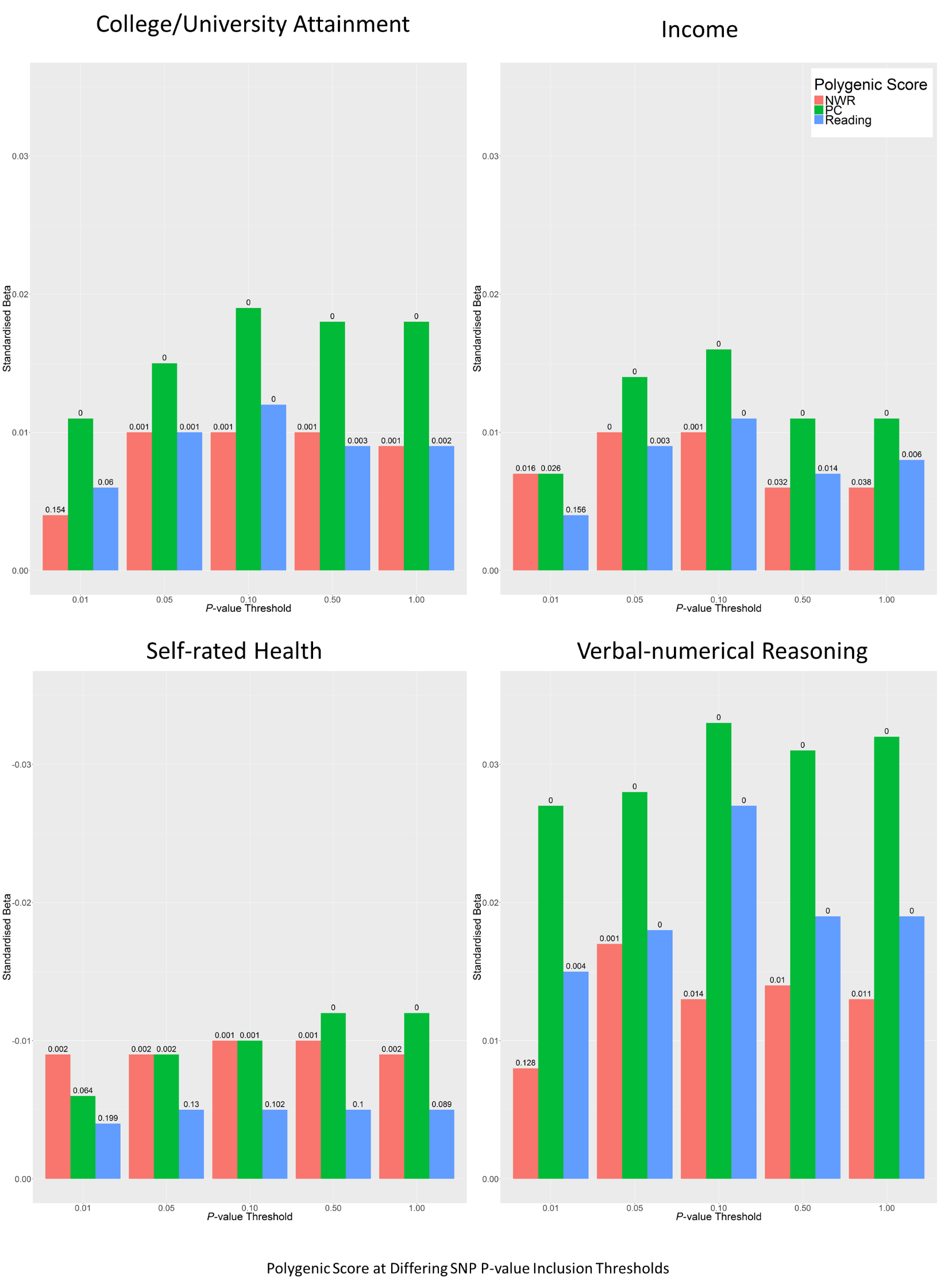

Supplement: Supplementary file 3 — Supplementary material 3 (DOCX 348 KB) [file 10519_2017_9859_MOESM3_ESM.docx]
